# Dual resistance of transgenic plants against *Cymbidium mosaic virus* and *Odontoglossum ringspot virus*

**DOI:** 10.1038/s41598-019-46695-7

**Published:** 2019-07-15

**Authors:** Ting-Yu Chen, Hsuan Pai, Liang-Yu Hou, Shu-Chuan Lee, Tzu-Tung Lin, Chih-Hao Chang, Fu-Chen Hsu, Yau-Heiu Hsu, Na-Sheng Lin

**Affiliations:** 1 Institute of Plant and Microbial Biology, Academia Sinica, 11529 Taiwan; 2 Graduate Institute of Biotechnology, National Chung Hsing University, Taichung, 40027 Taiwan

**Keywords:** Molecular engineering in plants, Virus-host interactions

## Abstract

Taxonomically distinct *Cymbidium mosaic potexvirus* (CymMV) and *Odontoglossum ringspot tobamovirus* (ORSV) are two of the most prevalent viruses worldwide; when co-infecting orchids, they cause synergistic symptoms. Because of the huge economic loss in quality and quantity in the orchid industry with virus-infected orchids, virus-resistant orchids are urgently needed. To date, no transgenic resistant lines against these two viruses have been reported. In this study, we generated transgenic *Nicotiana benthamiana* expressing various constructs of partial CymMV and ORSV genomes. Several transgenic lines grew normally and remained symptomless after mixed inoculation with CymMV and ORSV. The replication of CymMV and ORSV was approximately 70–90% lower in protoplasts of transgenic lines than wild-type (WT) plants. Of note, we detected extremely low or no viral RNA or capsid protein of CymMV and ORSV in systemic leaves of transgenic lines after co-infection. Grafting experiments further revealed that CymMV and ORSV trafficked extremely inefficiently from co-infected WT stocks to transgenic scions, presumably due to RNA-mediated interference. This study reports the first successful creation of dual resistant transgenic lines against CymMV and ORSV. Our studies shed light on the commercial development of transgenic orchid production to combat the global viral threat.

## Introduction

The Orchidaceae is the largest and most diverse family of flowering plants, about 28,000 species, distributed in about 763 genera^1^. Because of the fascinating array of colors, delicate flower shapes, and fragrant blooms, orchids have become popular and valuable ornamental plants in the market. However, orchids are susceptible to virus infection; at least 50 different viruses have been reported^2^. Of these, *Cymbidium mosaic virus* (CymMV) and *Odontoglossum ringspot virus* (ORSV) are the two most prevalent and economically important viruses affecting orchids worldwide^2^.

CymMV usually causes mild mosaic chlorotic or necrotic sunken patches on leaves and necrosis on flowers, whereas ORSV induces chlorotic ringspots or necrosis in leaves and color breaking and/or distortion on flowers. However, sometimes orchids infected by either of the two viruses do not display any symptoms but still show reduced vigor, growth rates and flower quality. Mixed infection with CymMV and ORSV commonly occurs worldwide and induces synergistic severe symptoms, including necrotic ringspots in leaves and mottling, ridging, curling and distortion of flowers^3–5^, leading to diminished flower size, quality and great economic loss.

CymMV, a *Potexvirus* member, has a single-stranded (ss) and positive-sense (+) RNA genome of about 6200 nucleotides (nts) consisting of five open reading frames (ORFs) and encoding a 160-kDa putative RNA-dependent RNA polymerase (RdRp) for viral replication, an overlapped tripe gene block (TGB) proteins (26, 13 and 10 kDa for TGBp1, TGBp2 and TGBp3, respectively) for movement and an 24-kDa capsid protein (CP)^6,7^. ORSV is a *Tobamovirus* member and has a ss, (+) RNA genome of about 6600 nts consisting of four ORFs and encoding a 126- and 183- readthrough RdRps, a 33-kD movement protein (MP) and an 18-kDa CP^8^. The CPs of CymMV and ORSV are expressed from their respective subgenomic RNAs. In addition, the CP of *Potexvirus* has been found required for cell-to-cell movement^9^, whereas that of *Tobamovirus* is a host-specific determinant of long-distance movement in plants^10^.

With mixed infection of CymMV and ORSV, the synergism of CymMV and ORSV in replication has been observed at the single-cell level in orchid protoplasts^4^. In addition, the complementation of MPs and CPs of CymMV and ORSV in cell-to-cell movement has been reported^3^. Although no insect vector has been reported in the field, we lack efficient measures to control virus diseases as compared with other pests and pathogens.

Pathogen-derived resistance has become one of the most powerful strategies to control virus diseases^11^. Various transgenes have been used successfully to confer virus diseases, including viral protein genes (replicase, MP or CP genes), viral RNAs (sense-, antisense- or double-stranded RNAs), satellite RNAs, defective interfering RNAs, hairpin RNAs, artificial microRNAs (miRNAs), host-derived resistance genes, or various factors involved in host defense responses (review in^12^). Previously, CymMV CP expression in transgenic *Nicotiana benthamiana*^13^, *Dendrobium*^14^ and *Phalaenopsis*^15,16^ could successfully protect plants against CymMV infection. Recently, Petchthai *et al*. (2018) using *Oryza sativa* miR528 as a backbone, expressed artificial miRNA (amiRNA) targeting replicase genes of CymMV and ORSV. AmiRNA transgenic *N. benthamiana* lines conferred high resistance to CymMV infection but weak resistance to ORSV infection^17^. No transgenic plants completely resistant to both CymMV and ORSV have been reported.

In this study, we used *N. benthamiana* as a model plant to express various constructs of CymMV and ORSV CP genes. Resistant transgenic lines showed a great reduction in CymMV and ORSV replication at the single-cell level as well as weak long-distance trafficking. This first successful example of dual resistance to CymMV and ORSV infection could be used to generate transgenic orchids.

## Results

### Plant transformation and transgene expression analysis

**Figure 1:**
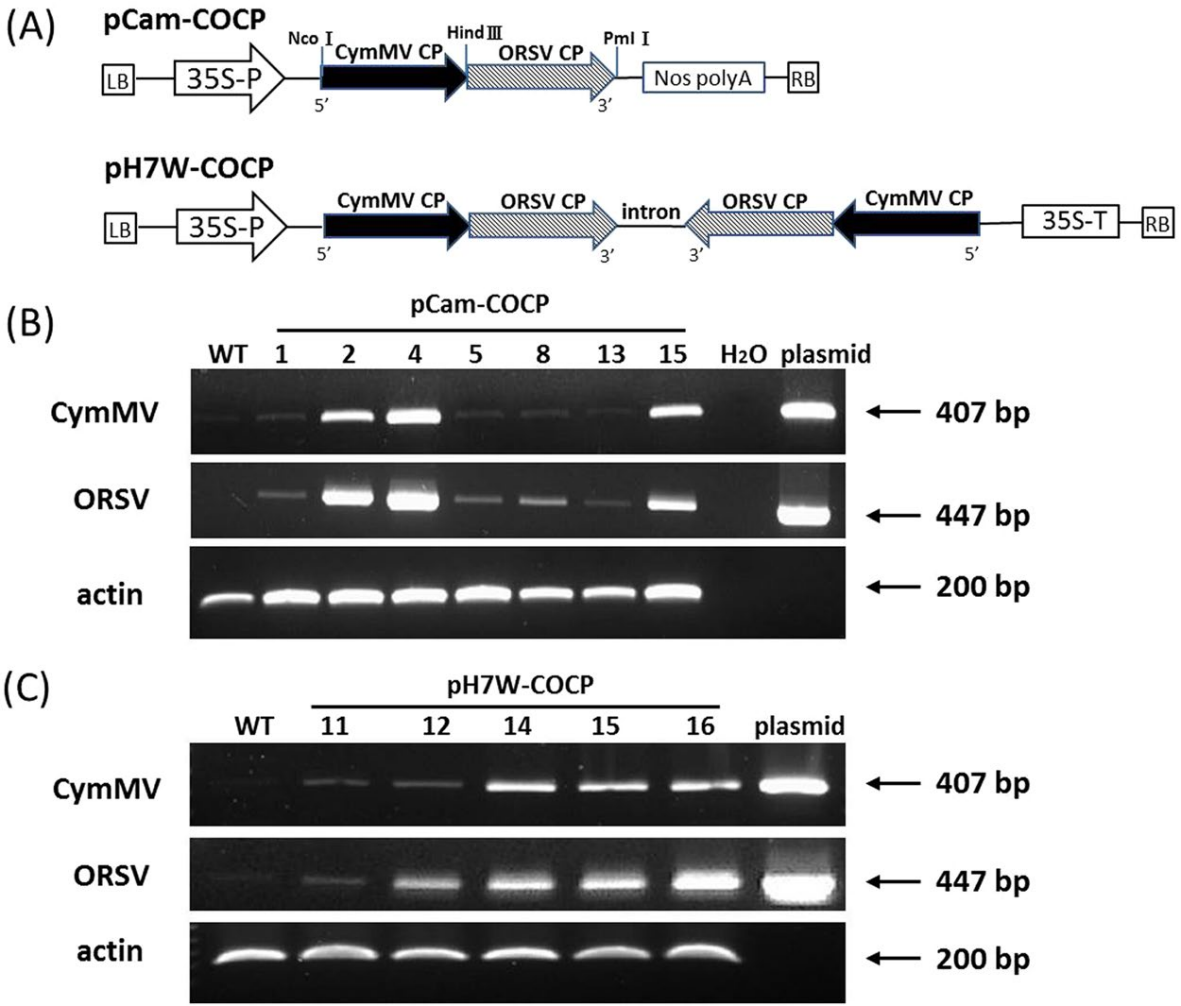
Physical map and analyses of transgenes in transgenic lines. (**A**) Schematic representation of pCam-COCP and pH7W-COCP constructs used for *N. benthamiana* transformation. (**B**,**C**) RT-PCR analysis of transgene expression in WT and transgenic pCam-COCP lines (**B**) and pH7W-COCP lines (**C**). The CymMV CP transcripts were detected by primer pair CyCP3 and CyCP6 with an expected size of 407 bp and ORSV CP transcripts by primer pair ORSV3 and ORSV4 of 447 bp. Plasmids pCam-COCP and pH7W-COCP were used for positive controls. The numbers on top of gel tracks indicate different transgenic lines. H_2_O and actin are negative and internal controls, respectively.

Leaf discs of *N. benthamiana* were transformed with *Agrobacterium tumefaciens* strain GV3101 containing pCam-COCP or pH7W-COCP; schematic representations of plasmid constructs are shown in Fig. 1A. Initially, embryogenic calli were induced and selected on medium containing hygromycin to regenerate putative T0 transgenic lines. Seedlings exhibiting resistance to hygromycin were examined for transgene expression by reverse transcription-polymerase chain reaction (RT-PCR). The expected product sizes of 407 and 477 base pairs (bps) for CymMV and ORSV, respectively, were obtained from *N. benthamiana* lines containing pCam-COCP or pH7W-COCP (Fig. 1B,C). We obtained a total of 7 independent T2 homozygous lines from pCam-COCP and 13 lines from pH7W-COCP after segregation analysis.

### Assessment of CymMV and ORSV resistance in transgenic *N. benthamiana* plants

**Figure 2:**
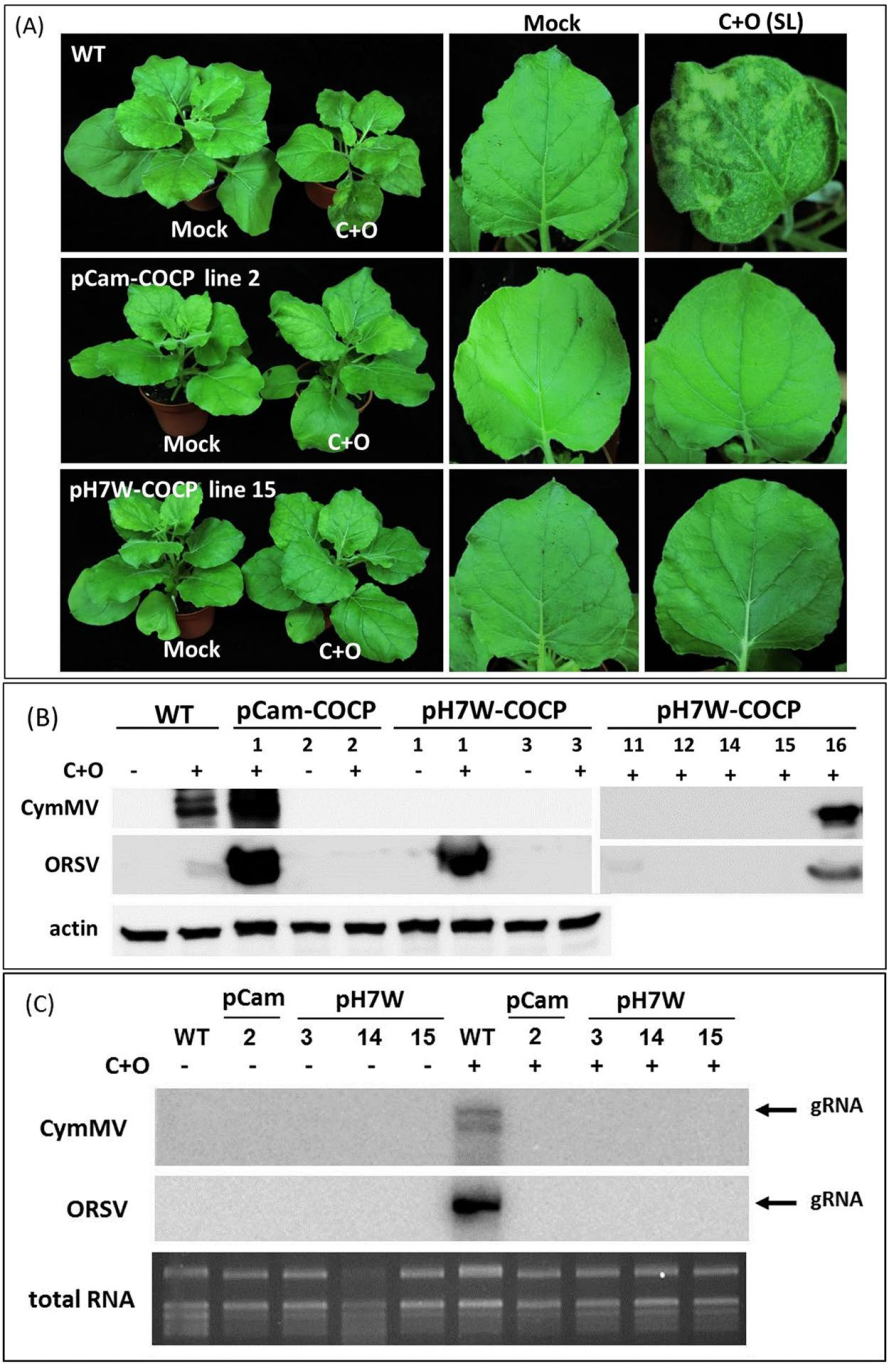
Assessment of CymMV and ORSV resistance in transgenic *N. benthamiana* plants. (**A**) Symptoms of WT and transgenic pCam-COCP line 2 and pH7W-COCP line 15 after inoculation with CymMV and ORSV virions (C+O). Photos were taken for whole plants and systemic leaves (SLs) at 20 dpi. (**B**) Western blot analysis of CymMV and ORSV capsid protein (CP) accumulation in SL of WT and transgenic lines inoculated with C+O at 20 or 26 dpi by antibodies against CymMV or ORSV CP, respectively. Actin was an internal control. (**C**) Northern blot analysis of viral RNA accumulation in WT and transgenic lines at 20 dpi with ^32^P-CTP labeled replicase probes for CymMV and ORSV. total RNA: EtBr staining of total RNA for equal loading. −: mock; +: C+O infection; gRNA: genomic RNA.

To examine whether these *N. benthamiana* transgenic lines were resistant to viral infection, all RT-PCR positive lines were individually inoculated with an equal amount of CymMV and ORSV virions (C+O). At 20 days post-infection (dpi), only transgenic line 2 expressing pCam-COCP (designated pCam-COCP line 2 hereafter) showed no symptoms on systemic leaves (SL), but other lines showed mottling, ridging, curling on inoculated leaves (IL) and SL, with stunted plant growth, as shown in the wild type (WT) plants (Fig. 2A). However, among the 13 transgenic lines expressing pH7W-COCP, several lines, such as 3, 14 and 15, exhibited no symptoms at 20 dpi after viral challenge (Fig. 2A).

We further performed enzyme-linked immunosorbent assay (ELISA) to measure the accumulation of CymMV and ORSV in WT and transgenic lines after C+O infection. As expected, the IL and SL of WT plants showed high ELISA values (>1.2) with C+O infection at 10 and 20 dpi, respectively. However, WT-mock-inoculated plants always showed low ELISA absorbance (<0.2), which can be considered a background level (Table 1). For transgenic lines expressing pCam-COCP, all lines had a slightly greater ELISA value (>2.0) than WT in IL for CymMV, but showed a wide range in SL. A background level was detected in SL of lines 2 and 15 for CymMV, with a similar low value for ORSV in line 2 but not in line 15 (Table 1). Thus, transgenic pCam-COCP line 2 could harbor CymMV and ORSV accumulation in IL but restricted viruses from moving to SL. In contrast, lines 1, 4, 5, 8 and 13 were permissive to a high accumulation of CymMV and ORSV in SL at 20 dpi (Table 1).

**Table 1:** ELISA values (mean ± standard deviation) for *Cymbidium mosaic potexvirus* (CymMV) and *Odontoglossum ringspot tobamovirus* (ORSV) accumulation in *N. benthmiana* wild-type (WT) and transgenic lines after challenge with CymMV and ORSV virions.

Plants		Infection^a^	CymMV level^b^		ORSV level^b^		Resistance^c^	
	Line		IL^d^	SL^e^	IL	SL	CymMV	ORSV
WT		mock	0.16 ± 0.01	0.14 ± 0.01	0.18 ± 0.01	0.15 ± 0.01	−	−
		C+O	1.68 ± 0.19	3.34 ± 0.17	1.87 ± 0.06	1.23 ± 0.33	−	−
pCam-COCP	1	C+O	2.75 ± 0.16	3.24 ± 0.17	0.92 ± 0.10	1.31 ± 0.06	−	−
	2	C+O	1.94 ± 0.35	0.22 ± 0.07	0.20 ± 0.03	0.17 ± 0.01	+	++
	4	C+O	2.02 ± 0.12	2.38 ± 0.25	0.76 ± 0.14	0.90 ± 0.34	−	−
	5	C+O	2.00 ± 0.11	2.04 ± 0.16	0.82 ± 0.22	1.05 ± 0.19	−	−
	8	C+O	2.09 ± 0.15	2.15 ± 0.18	0.73 ± 0.18	0.78 ± 0.39	−	−
	13	C+O	2.39 ± 0.35	2.60 ± 0.20	0.84 ± 0.18	1.25 ± 0.30	−	−
	15	C+O	2.79 ± 0.07	0.14 ± 0.02	0.93 ± 0.07	1.62 ± 0.15	+	−
pH7W-COCP	1	C+O	2.69 ± 0.09	0.12 ± 0.01	0.89 ± 0.09	1.41 ± 0.12	+	−
	3	C+O	1.45 ± 0.08	0.15 ± 0.01	0.17 ± 0.01	0.14 ± 0.01	+	++
	7	C+O	2.58 ± 0.24	2.24 ± 0.08	0.79 ± 0.38	1.68 ± 0.80	−	−
	8	C+O	2.40 ± 0.17	0.18 ± 0.01	0.36 ± 0.02	1.07 ± 0.35	+	−
	9	C+O	2.67 ± 0.25	0.26 ± 0.10	0.77 ± 0.08	2.21 ± 0.22	−	−
	10	C+O	1.98 ± 0.05	0.17 ± 0.02	0.49 ± 0.07	1.25 ± 0.73	+	−
	11	C+O	2.28 ± 0.44	0.17 ± 0.01	0.74 ± 0.10	2.71 ± 0.09	+	−
	12	C+O	2.24 ± 0.41	0.20 ± 0.02	0.58 ± 0.17	0.86 ± 0.14	+	−
	13	C+O	2.75 ± 0.22	0.22 ± 0.04	0.89 ± 0.14	1.06 ± 0.54	−	−
	14	C+O	1.72 ± 0.11	0.18 ± 0.03	0.14 ± 0.07	0.20 ± 0.09	+	++
	15	C+O	1.43 ± 0.07	0.16 ± 0.03	0.11 ± 0.02	0.19 ± 0.10	+	++
	16	C+O	2.10 ± 0.27	2.90 ± 0.67	1.03 ± 0.27	3.03 ± 0.51	−	−
	17	C+O	2.73 ± 0.21	2.08 ± 0.07	1.36 ± 0.24	3.37 ± 0.39	−	−

^a^Mock: mock-inoculated; C**+**O: mechanical inoculation of mixed virions of CymMV (C) and ORSV (O).

^b^ELISA value: the OD (A405 nm) value showing virus accumulation in inoculated leaves (IL) and systemic leaves (SL) after inoculation with C**+**O, which was obtained from three individual samples of each plant type.

^c^“−”: ELISA value > 2.0; “+”: ELISA value for IL > 0.8, SL < 0.2; “++”: ELISA value < 0.2 in both IL and SL.

^d^IL: inoculated leaves at 10 dpi.

^e^SL: systemic leaves at 20 dpi.

For transgenic lines expressing pH7W-COCP, CymMV detected in SL and ORSV in IL and SL of lines 3, 14 and 15 were close to WT-mock level of ELISA values (<0.2) (Table 1 and Fig. S1), so these three lines could be resistant to C+O co-infection. Among other lines, the IL of those lines showed higher ELISA value for CymMV and ORSV (both > 0.2) than the WT-mock level. Especially, the SL of lines 1, 8, 10, 11, 12 showed only low values for CymMV but high values for ORSV, which suggests that the five lines might be resistant to only CymMV but not ORSV. No other lines showed resistance to only ORSV (Table 1). Collectively, the efficiency of generate resistant lines from pH7W-COCP construct is higher than that from pCam-COCP. Moreover, differential resistance against CymMV and ORSV was noted between transgenic lines.

To further confirm the viral resistance in transgenic lines, we performed Western and Northern blot analyses. When the selected resistant transgenic lines described above were co-inoculated with C+O, on Western blot analysis, CymMV and ORSV CPs were not detectable in pCam-COCP line 2 with antibodies specific to CymMV or ORSV (Fig. 2B) nor in transgenic pH7W-COCP lines 3, 12, 14, and 15 at 20 or 26 dpi (Fig. 2B). Conversely, for lines showing severe symptoms after C+O co-inoculation, CymMV and ORSV CP levels were substantial in transgenic pCam-COCP line 1 and pH7W-COCP line 16, but pH7W-COCP line 1 and 11 were infected with ORSV only (Fig. 2B), which is consistent with ELISA results (Table 1 and Fig. S1). On Northern blot analysis, genomic RNAs of both CymMV and ORSV were again undetectable in resistant transgenic lines at 20 dpi (Fig. 2C), regardless of extraction from plants with or without C+O co-inoculation. All results indicate that transgenic pCam-COCP line 2 and pH7W-COCP lines 3, 14 and 15 are highly resistant to C+O co-infection.

### Virus accumulation is greatly reduced in protoplasts from resistant transgenic plants

**Figure 3:**
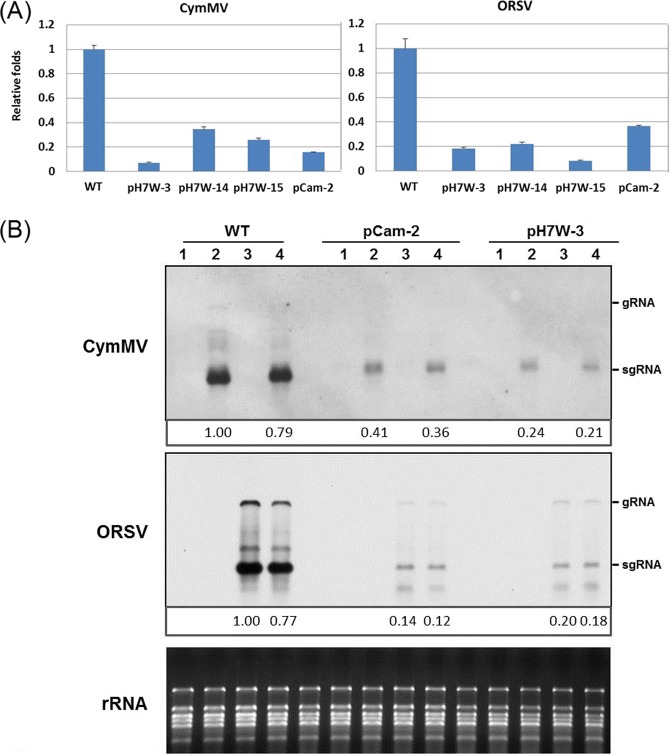
CymMV and ORSV RNA accumulation in protoplasts from WT and transgenic lines. (**A**) RT-qPCR analysis of CymMV and ORSV RNA in protoplasts of WT and transgenic lines after C+O infection at 18 hpi. Values are normalized against CymMV and ORSV RNA in protoplasts of WT plants infected with C+O. (**B**) Northern blot analysis of CymMV and ORSV in protoplasts from WT and transgenic lines transfected with C+O by DIG-labeled CP probes. Protoplasts were mock-inoculated with H_2_O (Lane 1), CymMV virions (Lane 2), ORSV virions (Lane 3), and mixed C+O (Lane 4). The relative accumulation of CymMV and ORSV in transgenic lines were shown compared with single virion inoculation in WT plants.

Successful virus infection involves several important steps: virus replication, cell-to-cell movement and long-distance movement in hosts. To determine whether the virus resistance occurred at the single-cell level, protoplasts were isolated from WT and transgenic lines and inoculated with C+O mediated by polyethylene glycol (PEG). On quantitative reverse transcription polymerase chain reaction (RT-qPCR) analysis, the accumulation of CymMV in protoplasts of transgenic lines, such as pCam-COCP line 2 and pH7W-COCP lines 3, 14 and 15, decreased from 63% to 92% as compared with the WT; ORSV accumulation decreased from 62% to 90% (Fig. 3A). Northern blot analysis also confirmed the reduced CymMV and ORSV RNA levels in transgenic protoplasts of pCam-COCP line 2 and pH7W-COCP line 3 (Fig. 3B). Although protoplasts of transgenic lines could be infected with CymMV and ORSV, only low replication efficiency of CymMV and ORSV were observed.

### Similar cell-to-cell movement efficiency between the leaves of WT and transgenic lines

Given that the accumulation of CymMV and ORSV in protoplasts and IL was significantly lower in resistant transgenic lines than WT plants and almost no virus was detectable in SL of transgenic lines (Table 1 and Fig. S1), we considered that viral trafficking may be limited in transgenic lines. Therefore, we tested whether cell-to-cell movement was affected in transgenic lines by using eGFP or 2x.eGFP as reporters in bombardment assays. Leaves of WT and transgenic lines were bombarded with pCass-eGFP to assess cell-to-cell trafficking efficiency. Confocal microscopy revealed free eGFP fluorescence localized in single cells (65.2%) in more than half of the observed sites in the WT at 40 h post-bombardment; however, eGFP also moved to two or three cells at the sustainable level (>30%) (Table 2 and Fig. S2). Transgenic pH7W-COCP line 3 showed similar or slightly higher trafficking efficiency of eGFP (Table 2 and Fig. S2B). To create a larger fluorescent marker, we fused two tandem copies of eGFP to generate pCass-2x.eGFP. Although 2x.eGFP was restricted in nearly 80% of observed sites, WT and transgenic pH7W-COCP line 3 showed no significant difference in 2x.eGFP movement beyond more than 2 cells (Table 2 and Fig. S2C,D). Hence, the gating of plasmodesmata (PD) for cell-to-cell movement was similar in transgenic line and the WT.

**Table 2:** Cell-to-cell movement of eGFP and 2x.eGFP in leaves of *N. benthamiana* WT and transgenic pH7W-COCP line 3 at 40 hours after particle bombardment.

	WT		pH7W-COCP line 3
eGFP	1 cell	43/66 (65.2%)	53/65 (81.5%)
	2 cells	15/66 (22.7%)	10/65 (15.5%)
	>2 cells	8/66 (12.1%)	2/65 (3.0%)
2x.eGFP	1 cell	38/51 (74.5%)	46/57 (80.7%)
	2 cells	10/51 (19.6%)	8/57 (14.0%)
	>2 cells	3/51 (5.9%)	3/57 (5.3%)

### Long-distance movement of CymMV and ORSV was impeded in transgenic line

**Figure 4:**
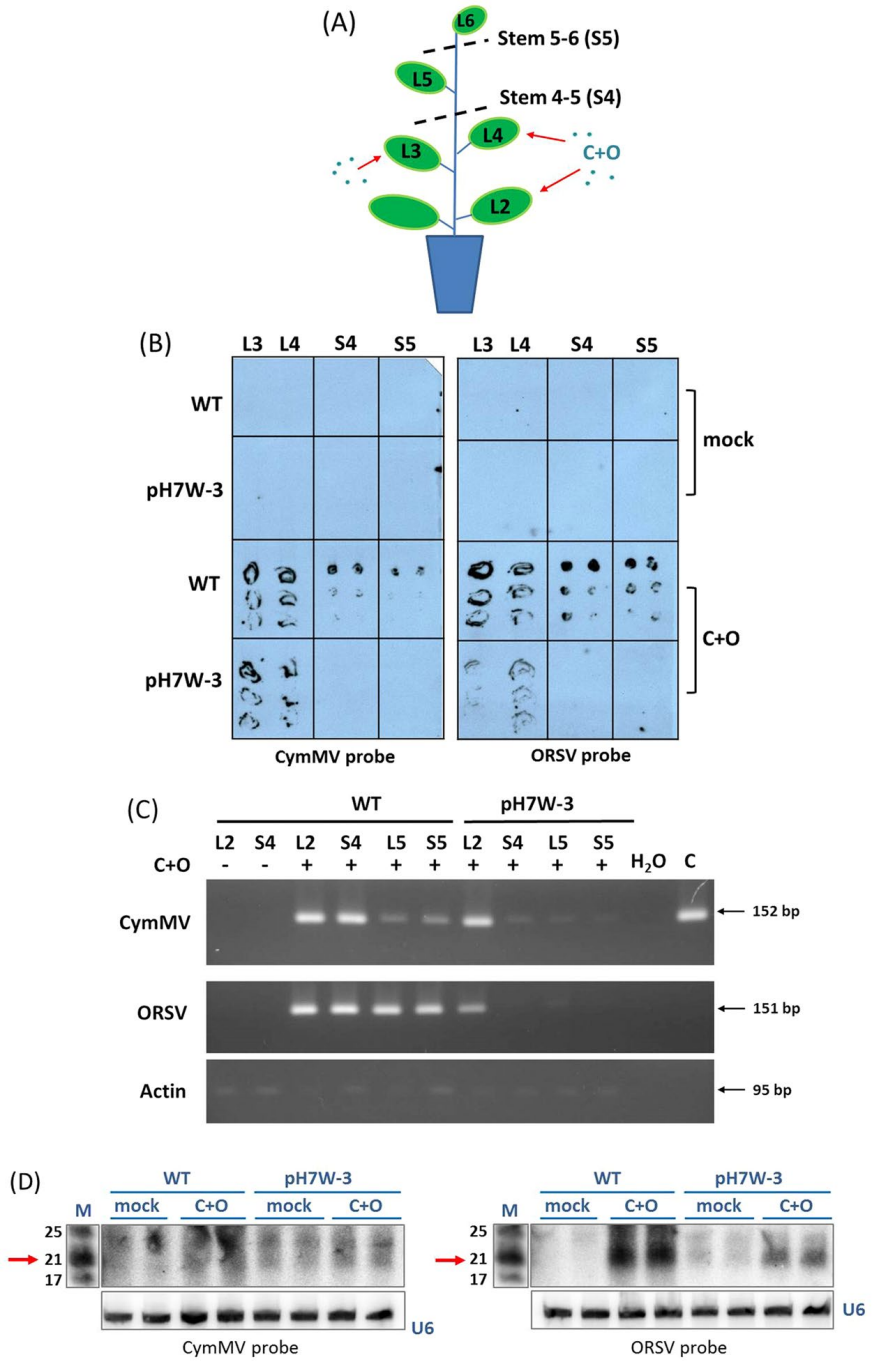
CymMV and ORSV viral RNA and siRNA accumulation in WT and transgenic plants inoculated with C+O. (**A**) Illustrations of sampling tissues in WT and transgenic line pH7W-COCP line3 (pH7W-3) after Leaf 3 (L3) and leaf 4 (L4) co-inoculated with C+O. L3, L4, Stem between L4 and L5 (S4), Stem between L5 and L6 (S5) and systemic leaf 5 (L5) were harvested for tissue blotting (**B**) and RT-PCR (C) at 14 dpi. (**B**) The tissue blots prepared from various tissues of WT and transgenic line were hybridized with DIG-labeled CymMV- and ORSV-specific CP probes. (**C**) Accumulation of CymMV and ORSV viral RNA in L2, S4, S5 and L5 of WT and transgenic line by RT-PCR at 14 dpi. CymMV RNA was detected by primer pair (CymMV RdRp-F and CymMV RdRp-R) of expected size 152 bp and ORSV RNA by primer pair (ORSV RdRp-F and ORSV RdRp-R) of 151 bp. Actin was used for loading controls. (**D**) Detection of the vsiRNAs in mock and C+O infected IL of WT and transgenic pH7W-3 at 10 dpi by [γ-^32^P] CTP-labeled CymMV or ORSV CP probes. The lower panel shows hybridization of U6 probes as equal loading control. The positions of 21 nt RNA is indicated by arrows. M: marker; mock: water inoculation; C+O: inoculated with CymMV and ORSV.

To further determine whether the long-distance trafficking of the viruses was affected, we measured virus systemic movement in the WT and transgenic pH7W-COCP line 3 (pH7W-3) by tissue blotting analysis from rolled IL and stems harvested at 14 dpi. We found strong hybridization signals for CymMV and ORSV RNAs in IL, e.g. leaf 3 (L3), leaf 4 (L4) and stems between inoculated L4 and non-inoculated leaf L5 (S4) or stems between upper non-inoculated L5 and leaf L6 (S5) of the WT (Fig. 4A,B). In contrast, weak signals for ORSV RNA were obtained in the IL of transgenic pH7W-3, in agreement with ELISA results (Fig. S1). However, we found no detectable signals in both stems of S4 and S5 in transgenic pH7W-3 with CymMV or ORSV probes (Fig. 4B), indicating that the long-distance movement of CymMV and ORSV might be blocked in the transgenic line. To confirm the detection, we carried out a more sensitive assay such as RT-PCR. With this procedure, we detected CymMV and ORSV viral RNAs in both WT and transgenic pH7W-3, but a sharp difference in an extremely weak signal in non-inoculated leaf L5, S4 or S5 in transgenic line compared with the inoculated WT plants. These results further confirmed that CymMV and ORSV confined in the ILs with trafficking from IL to SL in an inefficient manner in resistant line.

**Figure 5:**
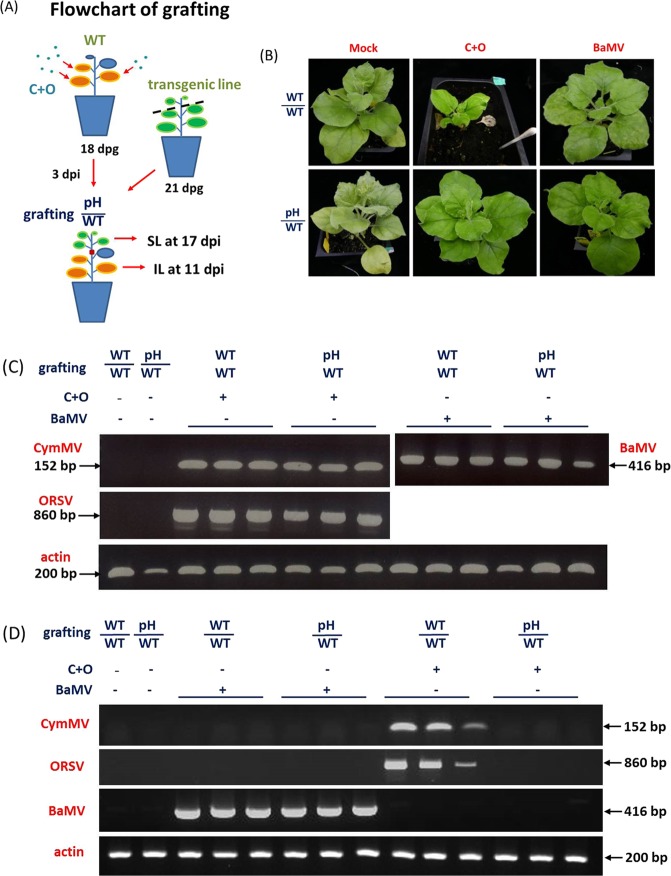
Long-distance movement of CymMV and ORSV in WT and transgenic pH7W-COCP line 3 (pH) by grafting assay. (**A**) Flowchart of grafting. (**B**) Symptoms of grafting plants inoculated with C+O at 17 dpi. (**C**,**D**) RT-PCR analysis of CymMV and ORSV RNA accumulation in grafted stock leaves at 11 dpi (**C**) and systemic scion leaves at 17 dpi (**D**). CymMV was detected by primer pair (CymMV RdRp-F and CymMV RdRp-R) of 152 bp; ORSV by primer pair (ORSV-RdRpPai-F and ORSV-RdRpPai-R) of 860 bp and BaMV by primer pair (BaMV-P2-F and BaMV-P2-R) of 416 bp, respectively. Actin was an internal control.

Next, to explore the long-distance trafficking of CymMV and ORSV in transgenic lines again, we grafted transgenic pH7W-3 onto virus-infected WT plants (pH/WT). WT plants were first co-inoculated with C+O and served as stocks at 3 dpi, with transgenic pH7W-3 as scions (Fig. 5A). In parallel experiments, mock-inoculated WT scions or *Bamboo mosaic virus* (BaMV)-inoculated WT stocks served as controls. As expected, grafted control plants (WT/WT) with C+O infection showed severe leaf curling, chlorotic leaves and stunted growth at 17 dpi, which did not appear on grafted scion plants (pH/WT). In contrast, BaMV caused chlorotic and mosaic leaves on both grafted plants (WT/WT; pH/WT) (Fig. 5B).

CymMV and ORSV viral RNA accumulation was measured in IL of stocks at 11 dpi and scions at 17 dpi by RT-PCR (Fig. 5A). The accumulation of CymMV, ORSV or BaMV RNA was easily detected in stock leaves from grafted plants (pH/WT) or control plants (WT/WT) (Fig. 5C). However, CymMV and ORSV RNAs were nearly undetectable in grafted scion leaves (pH/WT) at 17 dpi (Fig. 5D). In contrast, BaMV RNA level was substantially detected in both grafted scions (WT/WT and pH/WT) at 17 dpi. Thus, CymMV and ORSV were not only limited in transport from WT stocks to transgenic scions, but such long-distance movement blocking were CymMV- and ORSV- specific.

### Virus-specific small interfering RNAs (vsiRNAs) production in transgenic line

To evaluate if the viral resistance in transgenic line was resulted from gene silencing, we detected CymMV- and ORSV- specific vsiRNAs in inoculated WT and transgenic lines. RNA samples of C+O co-infected or mock-inoculated leaves of WT and transgenic pH7W-3 at 10 dpi were hybridized with CymMV and ORSV probes by northern blot analyses. Both CymMV and ORSV probes showed much stronger signals in the WT-inoculated leaves than in the transgenic lines, predominantly in 21 nt. Interestingly, both probes also detected abundant vsiRNAs from mock-inoculated transgenic line, which was absent in the mock-inoculated WT plants (Fig. 4D), suggesting transgene-derived vsiRNA production could trigger gene silencing for viral resistance in transgenic line during infection.

## Discussion

In this study, we successfully generated transgenic *N. benthamiana* plants expressing two different constructs, pCam-COCP or pH7W-COCP (Fig. 1A), that were able to confer resistance to both CymMV and ORSV (Table 1). Of note, more resistant lines against the two viruses were generated from transgenic lines with the pH7W-COCP transgene (Fig. 1B,C), which has a hairpin structure containing the full-length CP genes of CymMV and ORSV under control of the 35S promoter. Our finding agrees with the notion that double-stranded RNA is a powerful inducer in RNA silencing than either sense or antisense RNA alone^18^. In addition to double resistant lines, more lines were solely resistant to CymMV than ORSV (Table 1). The Tobamovirus-encoded P126 protein has been shown as a strong virus suppressor of RNA silencing (VSR); it affects gene silencing dose-dependently and also increases the plant susceptibility to several viruses^19^. In contrast, TGBp1 encoded by Potexvirus is usually a weak VSR^20^. This phenomena may explain why we could generate more resistant lines to CymMV but few to ORSV or both in our study. Previously, different strategies for designing transgenes have been attempted, including using the sense or antisense CymMV CP gene^14,21^ and amiRNA targeting RdRp genes of CymMV and ORSV^17^, but most transgenic studies have successfully conferred resistance to only CymMV. To date, no transgenic lines with resistance to ORSV or efficient dual resistance to both viruses have been reported.

Among all the transgenic lines we generated, the highly resistant lines pCam-COCP line 2 and pH7W-COCP lines 3, 14 and 15 were selected after virus resistance assays. These transgenic lines showed multilayer resistance during virus infection. First, both the accumulation of CymMV and ORSV was approximately 80% less in protoplasts of resistant lines than WT plants (Fig. 3), which represents the low replication efficiency at the single-cell level in transgenic lines. This less efficient virus replication could also result in lower viral accumulation in SL, as for some Geminivirus^22^. However, viral RNAs were nearly undetectable in SL of inoculated transgenic lines with C+O by ELISA (Table 1 and Fig. S1), Western blot analysis (Fig. 2B), Northern blot analysis (Fig. 2C), and RT-PCR (Fig. 4C). These results may suggest that virus long-distance movement is another defense layer against dual virus infection in transgenic line, although the PD gating ability was similar to WT plants (Fig. S2).

Tissue blotting further confirmed the lack of virus accumulation in vascular systems of resistant lines (Fig. 4B). For virus infection, the phloem-bundle sheath interface is a crucial barrier to several viruses entering into phloem long-distance trafficking during the virus–host interaction; these viruses include *Grapevine berry inner necrosis virus*^23^, *Tomato aspermy virus*^24^, *Red clover necrotic mosaic virus*^25^ and *Cucumber mosaic virus*^26^. Hence, CymMV and ORSV may be restricted at the phloem-bundle sheath barrier. However, whether CymMV and ORSV are blocked at the parietal layer of the sieve element or whether CymMV and ORSV can invade phloem parenchyma cells, companion cells, or the sieve element through the bundle sheath in the IL of resistant transgenic lines remains to be determined.

In higher plants, RNA molecules including siRNAs, miRNAs, transfer RNAs, ribosomal RNAs and mRNAs could traffic via the phloem to distant tissues, which is essential for nutrient allocation, gene silencing, stress responses and plant development^27^. Grafting experiments revealed several *Arabidopsis* mobile RNAs^28^ as well as small RNAs, including miRNAs, endogenous siRNAs or vsiRNAs^29^, are able to transport to distant tissues non-autonomously. Our grafting results strongly support that CymMV and ORSV were restricted in infected WT stocks with transgenic lines expressing CymMV and ORSV CP genes as scions (Fig. 5); presumably CymMV- and ORSV-specific vsiRNAs play a major role in systemic silencing^30^. In contrast, with similar grafting, BaMV RNA trafficked efficiently toward transgenic scions (Fig. 5), so these vsiRNAs could determine the viral RNA specificity and trafficking efficiency for viral resistance in resistant lines. Several studies have also indicated that non-transgenic plants grafted onto resistant transgenic lines could result in transfer of resistance to scions, such as with *Prunus necrotic ringspot virus*^31^, *Tomato spotted wilt virus*^32^ and *Tobacco mosaic virus*^33^. In double scions grafted onto a rootstock previously infected with *Pepper golden mosaic virus*, the resistant intermediate tissue delayed virus movement from the infected rootstock to the susceptible apex^34^. Such resistance is highly associated with early degradation of viral RNA by an siRNA mechanism^12^.

Taken together, our studies demonstrate the nearly complete dual resistance to CymMV and ORSV in *N. benthamiana* transgenic plants without any growth penalty by RNA-mediated interference. This is the first report of the development of transgenic plants with double resistance to the two most prevailing viruses, CymMV and ORSV, that infect orchids. Our study provides the exciting prospect of using similar transgenes to generate resistant orchids.

## Methods

### Construction of plasmids

We generated two constructs of transgenes for plant transformation. First, ORSV CP fragment was cloned from ORSV-infecting orchid total RNA. The first strand cDNA was synthesized with specific primer ORSV-CP-R, followed by PCR amplification with primers ORSV-CP-F and ORSV-CP-R (Table S1). *Xho*I and *Kpn*I –digested PCR product of ORSV CP fragment was then purified and cloned into pkylx7 vector in the cognate sites to generate pkylx-ORSVCP. To construct pkylx-COCP, the CymMV CP sequence was amplified by using pCYCP-11^14^ as template with primers CyCP9 and CyCP10 (Table S1). The fragment was inserted upstream to ORSV CP fragment in pkylx-ORSVCP via *Hind*III to generate pkylx-COCP. The whole cassette including the 35S promoter, CymMV-ORSV CP fragment, and rbcs terminator was amplified by primers 35S-1 and rbcSR3 (Table S1). The amplicon was then ligated into pCambia1301 through *BamH*I and *Xba*I sites to generate pCam-COCP. Second, to construct pH7W-COCP, the CymMV-ORSV CP fragment was first amplified from pkylx-COCP by attB1-COCP and attB2-COCP primers (Table S1) and cloned into the entry vector pDONR^TM^221, using BP clonase. The COCP fragment was then transferred into binary vector pH7GWIWG2I in sense and antisense orientation by using Gateway LR recombination (Life technologies). The constructs pCam-COCP and pH7W-COCP were transformed into *A. tumefaciens* strain GV3101, respectively.

### Generation of transgenic plants

Leaf discs of *N. benthamiana* were transformed with *A. tumefaciens* strain GV3101 containing pCam-COCP or pH7W-COCP as previously described^35^, and hygromycin (20 mg/l) was used as a selection marker. Transgenic shoots regenerated from leaf discs of *N. benthamiana* were grown on Murashige and Skoog agar medium (Sigma-Aldrich) containing hygromycin. T0 plants were transferred to soil and generated seeds. Transgenic plants were verified by PCR with specific primers (for COCP1 and COCP2, amplified size 1170 bp; Table S1). Homozygous individuals were selected from T1 and T2 progenies by hygromycin resistance and confirmed by RT-PCR with specific primer pairs (CyCP3 and CyCP6; ORSV3 and ORSV4; Table S1) for CP transgenes.

### Plant growth, inoculation and grafting

All WT and transgenic lines were grown at 28 °C in a walk-in plant growth chamber under a 16-h-light/8-h-dark cycle with white light (about 220 *μ*mol/m^2^/s^1^). For each experiment, at least 4 plants from each transgenic line were co-inoculated with CymMV and ORSV virions (0.05 *μ*g eacg/leaf) by gently rubbing virions onto 1 to 3 true leaves. For each construct transformation, 7 to 17 putative independent lines were assayed for resistance. Symptoms were observed visually till 20 dpi. The IL and SL were harvested at 10 and 20 or 26 dpi, respectively.

Grafting was performed as described^36^ except that 18~21-day-old *N. benthamiana* plants were used. WT plants were first inoculated with a mixture of CymMV and ORSV or BaMV virions^37^ as a control, then transgenic plants as scions were grafted onto infected WT plants at 3 dpi. The grafted plants were then incubated in a walk-in growth chamber for harvesting IL from stocks at 11 dpi and SL from scions at 17 dpi, and symptoms on grafted plants were continuously observed.

### ELISA and western blot analysis

From WT and transgenic plants, total protein was extracted from IL and SL at 10 and 20 dpi, respectively, by using coating buffer (15 mM Na_2_CO_3_, 35 mM NaHCO3, pH 9.6). For ELISA, a protocol was modified from Lin *et al*.^38^ () to measure the accumulation of CymMV and ORSV. Briefly, 96-microwell plates (Immuno 96 MicroWell plate, Nunc) were coated with total protein, then treated with the primary rabbit antibody for CymMV-CP or ORSV-CP (1:10,000 dilution) and secondary antibody, goat anti-rabbit immunoglobulin G conjugated with alkaline phosphatase (Applied Biological Materials, BC, Canada) (1:5,000 dilution). After the addition of substrate (p-nitrophenyl phosphate, 1 mg/mL), the absorbance at 405 nm was recorded by using an ELISA reader (PowerWave HT Microplate Spectrophotometer, BioTek Instruments, VT, USA) for 10 to 30 min.

For western blot analysis, proteins extracted from 0.1 g leaf of each sample were separated by 12% SDS-PAGE and transferred onto PVDF blotting membranes (GE Healthcare) as described^14^. Membranes were blocked with 5% milk in 1X PBS Buffer (Omics Bio) and incubated with the primary antibody for CymMV-CP or ORSV-CP (1:10,000 dilution) at 4 °C overnight, followed by the goat anti-rabbit IgG-HRP (1:5,000 dilution) and Western ECL substrate (Bio-Rad Laboratories, CA, USA).

### RNA analysis

Total RNA was extracted from 0.1 g leaf tissues by using TRIzol (Invitrogen, CA, USA). To identify the transgene expression in the transgenic line, RT-PCR was used with specific primer pairs for the COCP transgene. An amount of 2 µg total RNA was reverse transcribed by using a SuperScript III kit (Life Technologies, CA, USA) with poly(T) primer. The primer pairs (CyCP3 and CyCP6; ORSV3 and ORSV4) (Table S1) were used to amplify 407-nt CymMV-CP and 447-nt ORSV-CP cDNA fragments. The RT-PCR products were monitored by electrophoresis on 1.5% agarose gel in TBE buffer (Tris-borate-EDTA) and visualized with SYBR Green I core reagent (Life Technologies).

To detect the accumulation of CymMV and ORSV in WT and transgenic line after infection or grafting, RNA was extracted from various tissues and reverse transcribed into cDNA as described above. The accumulation of CymMV, ORSV and BaMV was detected by RT-PCR with specific primer pairs (CymMV RdRpF and CymMV RdRpR; ORSV RdRp-F and ORSV RdRp-R; ORSV RdRpPai-F and ORSV RdRpPai-R; or BaMV-P2-F and BaMV-P2-R), respectively (Table S1).

For RT-qPCR, cDNA template was mixed with 2X SYBR Green PCR master mix (Applied Biosystems) with the Applied Biosystems QuantStudio 12 K Flex Real-Time PCR system (Life Technologies). Specific primers (CymMV RdRp-F and CymMV RdRp-R; ORSV RdRp-F and ORSV RdRp-R) (Table S1) were used to detect CymMV and ORSV. Normalization of CymMV and ORSV RNA accumulation was with Nb Actin gene expression amplified by Nb-actin-F and Nb-actin-R (Table S1).

For Northern blot analysis, RNA gel blot was analyzed as described^39^, except that the membrane was hybridized with a DIG-labeled full-length CP probes or [γ-^32^P] CTP-labeled replicase probes for CymMV (EF125180, 2404–3722 nt, 1319 bp) and ORSV (AY571290, 2411–3270 nt, 860 bp). For CymMV and ORSV vsiRNA detection, 10 *μ*g total RNA was loaded for Northern blot analysis. The blots were probed with [γ-^32^P] CTP-labeled, carbonate buffer-fragmented RNA transcripts corresponding to CP genes of CymMV (EF125180) or ORSV (AY571290). Probing of markers and other details were performed as previously described^40^.

### Protoplast isolation and virus inoculation

Protoplasts were isolated from WT and transgenic plants and inoculated as described^41^. Approximately 2 × 10^5^ protoplasts were transfected with 0.6 *μ*g each of CymMV and ORSV viral RNAs or virions by PEG method. Total RNA was extracted from protoplasts by using the Hybrid-R kit (Gene All Biotechnology, Korea) at 18 hours after transfection for analysis of viral RNA accumulation by RT-qPCR and Northern blot assay.

### Monitoring cell-to-cell movement efficiency

To test whether cell-to-cell movement efficiency was affected in transgenic plants and the WT, we used eGFP and 2x.eGFP as reporters. The gold particles were coated with plasmid DNA by using a CaCl_2_/spermidine precipitation technique^42^. An amount of 10 *μ*l plasmid DNA (1 *μ*g/*μ*l, pCass-eGFP or pCass-2x.eGFP), 100 *μ*l 2.5 M CaC1_2_, 40 *μ*l 0.1 M spermidine and 100 *μ*l sterile gold particles (60 mg/ml, 1.0 *μ*m diameter) were mixed with continuous agitation in 1.5 ml Eppendorf tubes, vortexed continuously for 1 min, then centrifuged (6000 rpm, 30 sec). The supernatant was removed and the pellet was resuspended in 200 *μ*l absolute ethanol two or three times. The gold particles were resuspended in 100 *μ*l absolute ethanol. Aliquots of 10 *μ*l gene particle solution for each shot were used with GDS-80 for bombardment (Wealtec Bioscience Co.). The bombardment was set up with helium pressure 50 pounds per inch, vacuum 3.5 kgf/cm^2^, and 7-cm target distance. Leaf segments from WT and transgenic plants were used for bombardment assay. The eGFP and 2x.eGFP expressions were observed periodically under a confocal laser scanning microscope (LSM880, Zeiss) after bombardment. Results are expressed as number of fluorescent cell units per bombardment. Each experiment was repeated three times.

### Tissue blotting

WT and transgenic plants were first inoculated with mixed CymMV and ORSV. Sections were cut from fresh leaves and stem tissues 14 dpi by hand using a new razor blade. Tissue blots were made by pressing the newly cut surface onto Hybond^TM^ -N^+^ nylon membranes (GE Healthcare Life Sciences, Little Chalfont, UK) as described^43^. The blots were then hybridized with DIG-labeled RNA probes specific to CymMV or ORSV RdRp gene sequences by using the DIG Nucleic Acid Detection Kit (Sigma-Aldrich, MO, USA).

## Supplementary information

Supplementary information
