# Supplementary material for: Dual resistance of transgenic plants against Cymbidium mosaic virus and Odontoglossum ringspot virus
Source: Sci Rep. 2019 Jul 15;9:10230. doi: 10.1038/s41598-019-46695-7 (PMC6629631; doi:10.1038/s41598-019-46695-7)

## Supplementary information

# Dual resistance of transgenic plants against *Cymbidium mosaic virus* and *Odontoglossum ringspot virus*

Ting-Yu Chen[1], Hsuan Pai[1], Liang-Yu Hou[1], Shu-Chuan Lee[1], Tzu-Tung Lin[1], Chih-Hao Chang[1], Fu-Chen Hsu[1], Yau-Heiu Hsu[2] and Na-Sheng Lin[1*]

[1]Institute of Plant and Microbial Biology, Academia Sinica, Taiwan 11529

[2]Graduate Institute of Biotechnology, National Chung Hsing University, Taichung, Taiwan 40027

[*]Corresponding author: Na-Sheng Lin

E-mail address: nslin@sinica.edu.tw

**Supplementary Table S1.** Primers used in this study. The primer size and predicted product size are shown.

| Primer | 5’-sequence-3’ | Length (bp) | Product length (bp) |
|---|---|---|---|
| CyCP3 | GAGAGCCCACTCCAACTCCAGCTGC | 25 | 407 |
| CyCP6 | CCCAGTTGGCGGGCGGATCGTTA | 23 | |
| CyCP9 | CACAATAAAGCTTATAACCATGGGAGAGC | 29 | 735 |
| CyCP10 | CCACACGCCTTATTAAGCTTGGCGTTTTTCAG | 32 | (*Hind*III sites) |
| 35S-1 | CGCGGATCCGCCCGGGGATCTCCTTTGCCCCAGA | 34 | 3258 |
| rbcSR1 | CGCGGATCCTGATGCATGTTGTCAATCAATTGGC | 34 | (*BamH*I sites) |
| ORSV3 | ATGTCTTACACTATTACAGACCCGTCTAAG | 30 | 447 |
| ORSV4 | TTAGGAAGAGGTCCAAGTAAGTCCAGACATCG | 33 | |
| attB1-COCP | ACAAGTTTGTACAAAAAAGCAGGCT | 25 | 1100 |
| attB2-COCP | ACCACTTTGTACAAGAAAGCTGGGT | 25 | |
| COCP1 | CATGCCATGGATGGGAGAGCCCACTCCAA | 29 | 1170 |
| COCP2 | CCTAACACGTGTTAGGAAGAGGTCCAAGTAA | 31 | |
| CymMV RdRp-F | TCTGGGCGATCGCATTCATGAGGTT | 25 | 152 |
| CymMV RdRp-R | CCACGTCAGGTTGAGACTTTACC | 23 | |
| ORSV RdRp-F | GTATCAAAGGAAAAGCGGAGATGTA | 26 | 151 |
| ORSV RdRp-R | ATCAAGTCCCTTTGGAATGTATAGA | 25 | |
| Nb-actin-F | GATGAAGATACTCACAGAAAGA | 22 | 200 |
| Nb-actin-R | GTGGTTTCATGAATGCCAGCA | 21 | |
| BaMV-P2-F | GCGCTGCAGGGATCCATGGACCAGCCT | 27 | 416 |
| BaMV-P2-R | TTCGAATTCGGATCCTTAGCATGGTGG | 27 | |
| ORSV-RdRpPai-F | ATTGGCGACGAGTGGCCGTC | 20 | 860 |
| ORSV-RdRpPai-R | TGCAACAAGAACGTGCGGTGA | 21 | |
| ORSV CPF | ATCGCTCGAGATGTCTTACACTATTACAGACCCGTCTAAG | 40 | 467 |
| ORSV CPR | ATCGGGTACCTTAGGAAGAGGTCCAAGTAAGTCCAGACATCG | 43 | |
| Nb-actin-QF | TACGAGCTGCCTGATGGACAA | 21 | 95 |
| Nb-actin-QR | GCTTCCATTCCGATCATTGAT | 21 | |

**Supplementary Figure S1.** ELISA analysis of CymMV and ORSV accumulation in *N. benthamiana* WT and transgenic lines. (A) Inoculated leaves (IL) at 10 dpi. (B) Systemic leaves (SL) at 26 dpi.

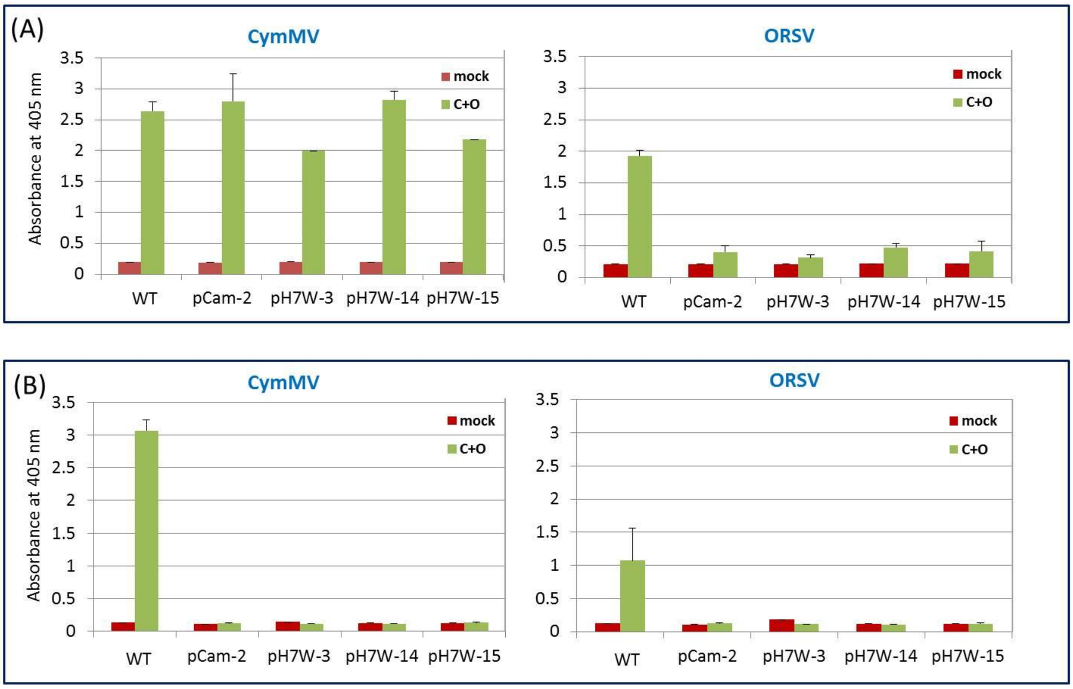

**Supplementary Figure S2.** Cell-to-cell movement of eGFP and 2x.eGFP in *N. benthamiana* leaves of WT and transgenic pH7W-COCP line 3 at 40 hr after particle bombardment. The eGFP in WT (A) and transgenic pH7W-COCP line 3 (B), 2x.eGFP in WT (C) and transgenic pH7W-COCP line 3 (D). 1: target cells showing eGFP or 2x.eGFP fluorescence. 2, 3, 4: eGFP or 2x.eGFP protein movement from target cell to the second, third or fourth cells.

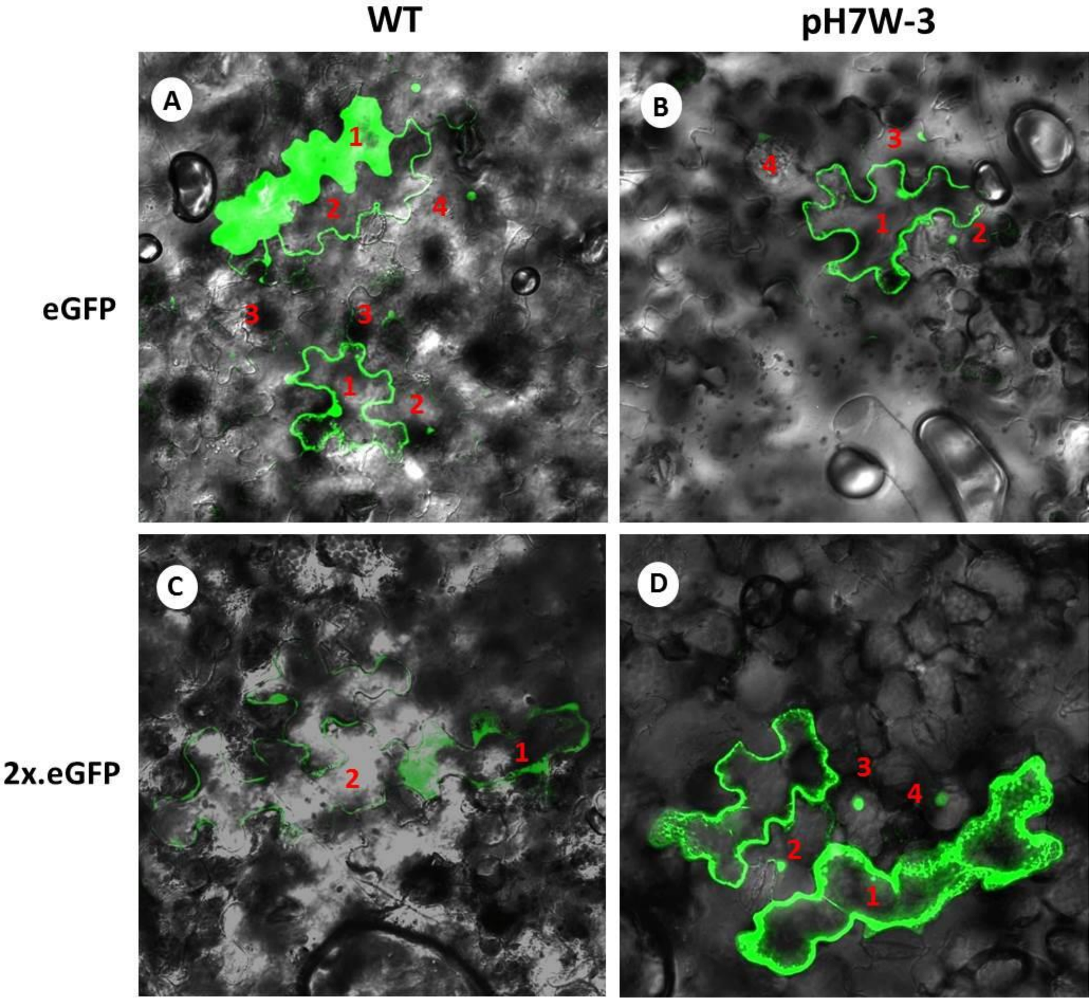

Supplement: Supplementary file 1 — Supplementary information [file 41598_2019_46695_MOESM1_ESM.pdf]
